# Calcifications of Vertebrobasilar Arteries on CT: Detailed Distribution and Relation to Risk Factors in 245 Ischemic Stroke Patients

**DOI:** 10.1155/2013/918970

**Published:** 2013-08-04

**Authors:** Slaven Pikija, Jožef Magdič, Tanja Hojs-Fabjan

**Affiliations:** Department of Neurology, University Medical Centre Maribor, Ljubljanska Cesta 5, 2000 Maribor, Slovenia

## Abstract

*Introduction*. Intracranial atherosclerosis is responsible for a substantial proportion of strokes worldwide but its detailed morphology in the vertebrobasilar arteries (VBA) is unknown. *Subject and Methods*. Cases with ischemic strokes were retrospectively sought from the hospital database. Native CT scans were assessed for vessel area and intracranial artery calcifications (ICACs) in VBA. The calcifications were classified as focal (FCs), crescent, and circular. *Results*. 245 patients (mean age: 77.1 ± 10.2 years, 57.6% females) had visible ICACs. Calcifications were found in 75.9%, 63.3%, and 17.1% in the left vertebral artery (LVA), the right vertebral artery (RVA), and the basilar artery (BA), respectively. FCs were present in 91.0%, 90.3%, and 100.0%; crescents in 30.3%, 29.0%, and 7.1%, and circulars in 6.4%, 4.8%, and 0.0% of the RVA, LVA, and BA, respectively. FCs in dorsolateral quadrant were least prevalent in both vertebral arteries (VAs): 46 (29.8%) and 46 (27.4%) for RVA and LVA, respectively. Risk factors associated with vertical dispersion of ICACs were male gender (OR : 2.69, 1.38–5.28) and diabetes (OR : 2.28, 1.04–4.99). *Conclusions*. FCs in VAs are least prevalent in dorsolateral quadrants. The vertical dispersion of ICACs seems to be associated with the male gender and diabetes.

## 1. Introduction

Intracranial atherosclerosis (ICAS) may be responsible for a substantial proportion of strokes worldwide [1]. Whereas ICAS is less prevalent in people of Caucasian ancestry, its frequency rapidly rises in people of Asian, African, and Hispanic backgrounds [2, 3]. In the stroke population of European ancestry, symptomatic ICAS is evenly distributed between brain vessels [4, 5]. Spotty calcifications in coronary arteries are linked to angina pectoris or infarction, whereas patients with stable angina tend to have larger calcifications [6, 7].

Calcification occurs in atherosclerotic plaque, is found in most people over 60 years, and tends to progress with ageing. It suggests the presence of plaque; however, plaques can be present without it. Apart from precipitated calcium deposits in necrotic tissue, calcifications may have “bone-like” structures driven by hematopoietic cells [8]. Animal data suggests that ICACs may be predisposed to plaque rupture by inducing its mechanical instability, but some disagree [9–11].

The cause of stroke in the territory supplied by VBA is most frequently atherosclerosis, and VAs are equally affected by the process with plaques rich with inflammatory substrates [9–13]. Atherosclerotic stenosis of VAs is most prevalent at the origin and in the intracranial (V4) portion, and their association with risk factors has already been investigated [14–17]. We have sought to determine a detailed distribution of calcifications in the intracranial portions of the vertebral arteries (V4 segment) and the basilar artery as seen on plain CTs in ischemic stroke patients. 

## 2. Subjects and Methods

The study was conducted at the Department of Neurology of the University Medical Centre Maribor, Slovenia, and was approved by the local ethics committee. We have retrospectively searched for consecutively admitted patients with stroke symptoms over a 12-month period. The search criteria were set for the admission period from September 2011 to October 2012. ICD-10 diagnoses were searched for the I63 code (ischemic stroke). Stroke was diagnosed by already established criteria (WHO criteria). A standard evaluation was performed. All patients had undergone ECG and CT examinations. A Doppler ultrasound of neck vessels, multislice CT angiography (CTA), MRI, digital subtraction angiography (DSA), heart ultrasound (transthoracic and/or transesophageal), and 24 h ECGs were performed in selected patients. The stroke neurologist (JM) collected variables from medical documentation: age, gender, previous TIA/stroke, and the presence of risk factors which were self-reported or present in medical charts, in particular arterial hypertension, atrial fibrillation, diabetes mellitus, myocardial infarction, heart failure, peripheral arterial occlusive disease (PAD), carotid artery stenosis over 50% or occlusion, previous procedures on neck arteries, an artificial heart valve, treatment with statins, and/or known hyperlipidemia. Since smoking history was not available in the medical records of almost 85% of patients, we have contacted the patient and/or the patient's family. All strokes (458 patients) were classified according to SSS-TOAST and OCSP based on clinical data available to the stroke neurologist (JM) [18, 19]. A CT was performed on two 64-slice multidetector CT systems.

The criteria for inclusion in the study were a native brain CT with 3 mm slices during hospitalization. The investigators (SP and JM) examined axial slices of the intracranial vertebral artery segment and the basilar artery. The measurement of HU density was performed in a CT window set at a width of 700 and a length of 250. The cut-off value of 90 HU was used for the presence of calcifications in the vessel wall [16, 20]. An imaginary template was drawn through the axial slice of vessel, dividing each vessel into four equal quadrants. For vertebral arteries (VAs) these were ventral medial (VM), ventral lateral (VL), dorsal medial (DM), and dorsal lateral (DL). For the basilar artery (BA) these were right ventral (RV), right dorsal (RD), left ventral (LV), and left dorsal (LD). Calcifications were coded as follows: (1) focal—involving no more than one quarter of the vessel circumference; (2) Crescent—extended over one quarter of the vessel circumference but not through the entire circumference; (3) circular—circular calcification along the entire vessel circumference. A vertical dispersion of calcifications was noted for every type of calcification; that is, the number of slices with present calcifications was counted. A number of distinct foci for focal calcifications were also noted. We have measured two vessel diameters (R1 and R2) perpendicular to each other on the same slice. To avoid a blooming effect, a measurement was carried out in the slice free of calcifications. In the BA, this was done right above the VAs junction. The area was calculated from these two diameters on the assumption that the vessel had an elliptical shape (R1 × R2 ×  Π). The calcifications were also distinguished according to their extension between contiguous slices as follows: “segmental”—present in one CT slice only; “continuous”—calcification at the same location was present in adjacent slices. Moreover, we made separate notes for the propagation of calcification from VAs to BA. The vessel was evaluated in the plane where it had the most circular cross-section, so the position of calcification was noted accordingly. At the same time, we have searched for the presence of intracranial carotid calcifications on either side. Previous studies have used various modifications of calcium scores [14, 16, 20]. Those scores were calculated from all the intracranial arteries. Since we were only interested in the VBA, the above mentioned scores were not suitable for our study. Morphological groups were formed according to various calcium distribution characteristics. A multifocal calcifications (MfCs) group was defined when two or more focal calcifications were present in VBA. The crescent and circular calcifications (CCC) group is the group with crescent and/or circular calcifications in any vessel. In order to discover how various types of calcifications coexist together, we have also analzsed groups with pure MfCs (PMfCs) and pure CCC (PCCC). PMfCs were defined as those without CCCs in any vessel. PCCC represents those CCCs without MfCs in any vessel. Patients were also grouped into those with only one FC to account for a “benign” pattern of calcification. Those were patients with a single FC in one slice and one vessel. In order to investigate a possible correlation between the horizontal (HDS) and the vertical dispersion (VDS) of ICACs, we have derived a dispersion score in two planes. The HDS was calculated as the total number of FCs in any vessel. An extra point was added if a crescent and/or a circular type of calcification were found. The VDS was calculated as the total number of slices affected by any type of calcification in any vessel. Again, an extra point was given if a calcification was present in at least two successive slices and another extra point if contiguous calcifications were present between the vertebral and basilar arteries. Smoking was dichotomized as “ever smoked” and “never smoked + unknown data.” STATA SE 11.2 was used for statistical analysis purposes. Moreover, we performed a Student's *t*-test for a mean comparison, Kendall's tau test with Bonferroni correction for the correlation of ordinal data, a chi-square test for categorical data, and logistic regression with a binomial outcome. A statistically significant difference was defined as the *α* level of .05 through the use of two-tailed tests.

## 3. Results

Over a 12-month period (from September 2011 to October 2012), there were 468 matches for ischemic stroke diagnosis (I63.*) in our medical system. Nineteen cases were excluded from the analysis, among which 1 patient had an intracerebral haemorrhage, 3 patients had CT scans with a slice thickness of over 3 mm, 6 patients did not have CT scans, 4 patients had only contrast CT scans available for review, 3 patients had only an MRI available, and, in 2 patients, excessive motion artefacts were present and the analysis was impossible. The demographics are provided in Table 1. Of 449 patients, the calcifications in any vessel were present in 245 (54.6%) patients and they are the focus of this report. An intracranial carotid calcification was present in 241 (98.4%) patients. The calcification distribution by calcification type is provided in Table 2. An unknown history was present in 9 (3.7%) patients. Smoking history was unknown in 61 (24.9%) patients. The male patients (*N* = 104, *M* = 73.4 ± 9.8 years) were younger than the female patients (*N* = 141, *M* = 79.9 ± 9.6 years) at *P* < 0.001. They also had a lower HDL (*M* = 1.19 ± 0.46 mmol/L versus *M* = 1.34 ± 0.47 mmol/L) at *P* = 0.039, higher triglycerides (*M* = 1.88 ± 1.34 mmol/L versus *M* = 1.52 ± 0.76 mmol/L) at *P* = 0.025, and higher creatinine levels (*M* = 97.7 ± 68.7 mmol/L versus 83.2 ± 40.1 mmol/L) at *P* = 0.051 and more frequently were smokers (41/104 versus 18/141 at *P* < 0.001). 

A brain MRI was performed in 17 (6.9%) patients. A precerebral ultrasound was performed in 85 (34.7%) patients, a 24 h ECG in 41 (16.7%), a heart ultrasound in 57 (23.3%), a cerebral CTA in 46 (18.8%), a precerebral CTA in 49 (20.0%), and a cerebral DSA in 4 (1.6%) patients. Creatinine values were available for 222 (90.6%) patients, cholesterol and triglycerides values were available in 194 (79.2%) patients, and LDL and HDL values were available in 188 (76.7%) patients.

The LVA artery had a significantly greater area than the right, 78.3 versus 70.9 mm^2^, respectively (*N* = 212, *P* = 0.006) and was dominant in 139 (56.7%) patients. The RVA was dominant in 99 (40.4%). Calcification of the RVA and BA was associated with a larger area (*N* = 225, *P* < 0.001 and *N* = 241, *P* = 0.021, resp.), but not in the LVA (*N* = 230, *P* = 0.318). When calcification was present in one artery only, it was present in the dominant VA at nearly the same percentage (66.7% and 62.0% for the LVA and RVA, resp., *P* = 0.487).

In patients with multifocal calcifications (MfCs) (*N* = 55, 22.4%), the treatment with statins (*N* = 20, 57.1%) was significantly more frequent than in those without MfCs (*N* = 40, 21.1%) at *P* = 0.020. The history of TIA (*N* = 7, 12.7%) and previous TIA/stroke (*N* = 21, 38.2%) was more frequent in patients with MfCs than in those without, at *P* = 0.035 and *P* = 0.05, respectively. Patients with MfC had greater odds of being treated with statins (OR = 2.29, 95% CI 1.13–4.65), even after being adjusted to age, gender, hypertension, previous TIA/stroke and diabetes.

In patients with CCC in any vessel (*N* = 84, 34.3%), the PAD (*N* = 9, 10.7%) was significantly more frequent than in those without CCC (*N* = 5, 3.1%) at *P* = 0.015. Diabetes mellitus was also more frequent in CCC patients, (*N* = 23, 27.4% versus *N* = 28, 17.4%); however, this difference did not affect the statistical significance (*P* = 0.068). Statin therapy was not associated with CCC pathology (*P* = 0.447). “Pure” MfCs (PMfCs) were few in number, only 29 (11.8%), and “pure” CCC (PCCC) was even scarcer, 19 (7.8%). PMfCs had more atrial fibrillation (AF) (*N* = 11, 21.6%) than in patients with another vessel pathology (*N* = 18, 9.3%), at *P* = 0.016. 

In 13 (5.3%) patients with PCCC (out of a total of 16), we observed an association with lower HDL (*M* = 1.03 ± 0.32 mmol/L versus *M* = 1.30 ± 0.47 mmol/L) at *P* = 0.052. 

There were 145 (59.2%) patients with VDS. Male patients had VDS more frequently (*N* = 75, 72.1%) than women (*N* = 70, 49.6%) at *P* < 0.001. Cholesterol was lower (*M* = 4.76 ± 1.30 mmol/L) than in patients without VDS (*M* = 5.16 ± 1.39 mmol/L) at *P* = 0.039. VDS patients had lower LDL values (*M* = 2.94 ± 1.12 mmol/L versus *M* = 3.27 ± 1.05 mmol/L) at *P* = 0.038. Statin therapy in VDS was more frequent (*N* = 43, 29.7% versus *N* = 17, 17.0%) at *P* = 0.024. In the multivariate model, the association with diabetes was shown to be an important predictor of VDS, as well as the male gender (Table 3).

The FCs were more frequently present among female patients (*N* = 42, 76.4%) than male patients (*N* = 13, 23.6%) at *P* = 0.001. The FCs were more frequently present among patients without diabetes (*N* = 49, 89.1%) than those with diabetes (*N* = 6, 10.9%) at *P* = 0.040. 

The median VDS was 3 (1–7) and the median HDS was 2 (1–4). The most prevalent combination of HDS and VDS was a 1-by-1 relationship, 62/245 (25.3%), and then a 2-by-2 pattern in 14/245 (5.7%). The HDS and VDS were correlated, *τ* = 0.62, *P* < 0.001, Figure 2. When the 1-by-1 combination was excluded, the association remained, *τ* = 0.57, *P* < 0.001.

## 4. Discussion

We have shown that calcifications in the VBA are not uniform regarding the site and type of calcifications as well as the affected vessel. Contrary to previous reports, which found an equal distribution of atherosclerosis in VAs, our data shows that the LVA is more frequently affected (76%) than the RVA (63%) [21, 22]. Greater vessel area was positively associated with calcification in the RVA and BA, but not in the LVA; also when calcification is present in only one VA, the dominant VA is more frequently calcified than its smaller counterpart. This observation could be caused by greater exposure of the dominant (and larger) artery to blood shear stress.

**Figure 1:**
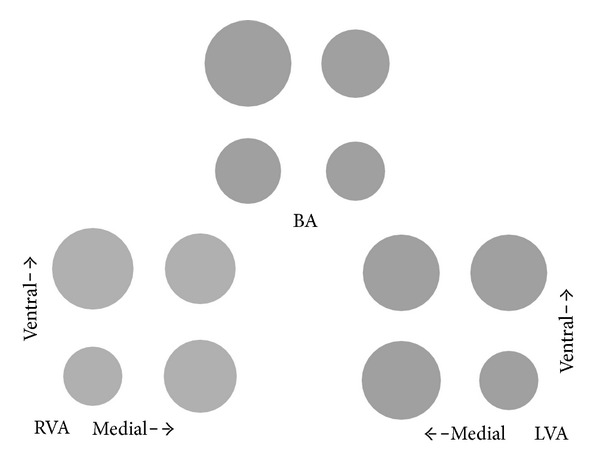
Horizontal distribution of focal calcifications in the vertebrobasilar arteries. The vertebrobasilar arteries with the relative distribution of focal calcifications. RVA—right vertebral artery; LVA—left vertebral artery; BA—basilar artery.

In both VAs (Figure 1) the DL quadrant is the least affected quadrant with focal calcifications. Perhaps this part of the vessel wall is less exposed to mechanical stress. 

Previous reports with an identical HU cut-off value cite 31.6–35.6% involvement of the vertebral arteries and 4.5–21.7% involvement of the basilar artery [16, 23]. We found a greater prevalence in the VA (52.4%) and a roughly similar prevalence in the BA (9.3%). The mean age of our entire population was 4 years older than that in the above mentioned studies, which might partially explain a higher prevalence of ICACs in our sample. Another recent study with a younger (*M* = 62–69.8 years) population also showed a smaller prevalence of ICACs, 14.0–37.3% for VAs and 0.0–3.5% for BA [14, 20]. The HU cut-off in both studies was set at 130 HU.

FCs can be regarded as a surrogate marker of an ongoing atherosclerosis and might represent the “tip of the iceberg” of the atherosclerotic plaque burden. Our data may point to the fact that FCs are developing consecutively and tend to progress to more elaborate calcifications as there are virtually no isolated “severe” calcifications (PCCCs), meaning that isolated crescent or circular calcifications virtually do not exist without simultaneous FCs. Recent studies showed that the progression of calcifications in carotid arteries is mostly dependent on the initial calcification load and the length of time passed [24]. Owing to the high prevalence of carotid artery calcification found in our cohort and the fact that these arteries are surrounded by bone structures, we did not evaluate these calcifications. 

In patients with ICACs, the BA is the least affected artery in only 17.1% of patients. The type of calcification observed in all samples was focal with only 7.1% crescent calcifications. Circular calcifications, which were observed in 4.8–6.4% of VAs, were not seen in the BA. It is not clear why there is such a difference in the types of calcification morphology; however, previous reports have also found a small prevalence of BA circular occlusive disease [25]. The ventral surface of the BA is affected by the focal type of calcifications in more than 90% of patients with ICACs, whereas the dorsal surface is calcified in only about 60% of patients. This is in line with previously reported observations on the greater prevalence of sudanophilic material in ventral portions (55%) apart from dorsal portions (43%) of the BA [26]. As the authors suggest, the flow parameters and curvature might play a role in the site of atherosclerosis in the BA. 

Male gender and diabetes history showed an association with VDS. Diabetes was previously shown to be associated with ICACs as well as with a greater extent of intracranial atherosclerosis and a higher risk of recurrent stroke and vascular death among Asian patients with symptomatic intracranial stenosis [16, 27, 28]. Apart from previous reports, age did not contribute to the risk of developing VDS in our sample, and the association with statin therapy after adjustment was lost. In addition, apart from previous reports that showed a positive association with hypertension and ICACs, we did not find such association between the ICACs groups [16, 23, 29]. As already reported, ICAS in our study was significantly more present in male patients than female patients [30]. In our study, male patients had an unfavourable risk profile with a lower HDL, higher TG, and smoking history. Male gender and a high TG are associated with coronary artery calcifications [31]. 

The history of statin therapy was positively associated with a horizontal dispersion of ICACs, but only of the multifocal type. The multifocal type of calcifications was also univariately associated with a history of TIA, but after the adjustment this association was lost. This finding could be paralleled with the fact that spotty calcifications tend to be more unstable and negatively affect plaque stability [6, 7]. Since the statin therapy was not associated with more advanced ICACs, we can speculate that the hyperlipidemia that prompted usage of statins already produced some irreversible changes that led to focal ICACs or propagation to crescent/circular ICACs. There is a remote possibility that the statin therapy has no effect on the occurrence of ICACs. Some reports even suggest that statins can be osteogenic [32, 33].

**Figure 2:**
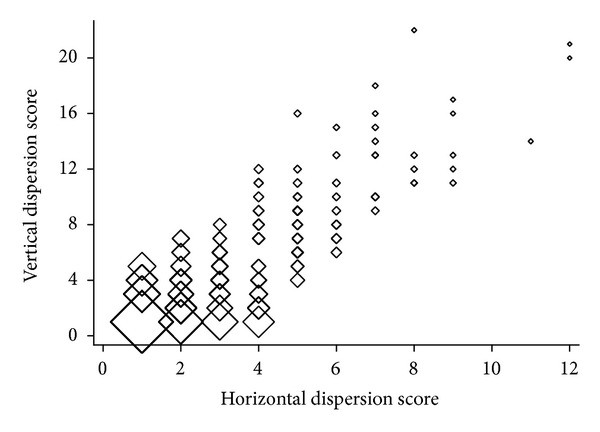
Correlation between the horizontal and vertical calcification dispersion scores. Data shows the frequency of the associated pairs of scores. Larger diamonds indicate more frequent pairs.

The association of the horizontal with the vertical dispersion of calcification was observed. The crescent and/or circular calcification types were more often vertically extended (Figure 2), whereas half of all focal calcifications were confined only to one CT slice. 

The etiology of the stroke was assessed with SSS-TOAST and OCSP classifications [18, 19]. Previous reports found that intracranial atherosclerosis is linked to large artery atherosclerosis or a small artery occlusion type of infarcts [29]. However, our data did not show any association between stroke etiology or location with either type of horizontal or vertical calcification distribution. 

The shortcomings of our study include its retrospective nature, modest number of patients, and somewhat incomplete evaluation of stroke etiology that could provide us with a better insight into the risk factor profile for different types and distributions of calcifications, as already reported in some studies [23]. Moreover, owing to the variable vascular anatomy and sometimes tortuous flow of VAs and BA, the examination of arteries with plain CT may not have been adequate in all patients. Also, our study was not designed to find the exact infarct distribution. We have not encompassed the third dimension, that is, calcification and/or plaque thickness. Calcifications in VBA arteries are more easily spotted on plain CT scans than the same pathology in the intracranial portion of the carotid arteries. Therefore the evaluation of calcifications in this territory on a CT could be more reliable. Furthermore, our study was not designed to interpolate clinical outcomes. However, the results, which were largely compatible with those of previous studies, give us reasonable strength to draw some valid conclusions.

In conclusion, our rather old ischemic stroke population showed that ICACs in both vertebral arteries and the basilar artery are highly prevalent. Vertical dispersion of ICACs seems to be linked with male gender and diabetes. Further studies are needed in order to expand the risk assessment to develop various morphological types of ICACs and to extend our findings. 

## Figures and Tables

**Table 1:** Demographic characteristics of ischemic stroke patients overall and grouped by vertically dispersed calcifications.

	All (*N* = 245)	No vertically dispersed calcifications (*N* = 100)	Vertically dispersed calcifications (*N* = 145)
Age (years)	79.0 (71.0–84.0)	78.0 (70.5–85.5)	79.0 (72.0–84.0)
Female	141 (57.6)	71 (71.0)	70 (48.3)
Prior stroke	54 (22.0)	21 (21.0)	33 (22.8)
Arterial hypertension	186 (75.9)	75 (75.0)	111 (76.6)
Diabetes mellitus	51 (20.8)	15 (15.0)	36 (24.8)
Atrial fibrillation	51 (20.8)	17 (17.0)	34 (23.4)
Heart failure	46 (18.8)	17 (17.0)	29 (20.0)
Statin therapy	60 (24.5)	17 (17.0)	43 (29.7)
Transitory ischemic attack	16 (6.5)	3 (3.0)	13 (9.0)
Previous TIA/stroke	68 (27.8)	23 (23.0)	45 (31.0)
Myocardial infarction	16 (6.5)	4 (4.0)	12 (8.3)
Peripheral arterial occlusive disease	14 (5.7)	4 (4.0)	10 (6.9)
Artificial heart valve	4 (1.6)	2 (2.0)	2 (1.4)
Carotid arteries disease	8 (3.3)	2 (2.0)	6 (4.1)
Carotid arteries intervention	3 (1.2)	1 (1.0)	2 (1.4)
Smoking history*	59 (24.1)	21 (21.0)	38 (26.2)
OCSP classification			
TACI	47 (19.2)	14 (14.0)	33 (22.8)
PACI	144 (58.8)	60 (60.0)	84 (57.9)
LACI	1 (0.4)	0 (0.0)	1 (0.7)
POCI	50 (20.4)	25 (25.0)	25 (17.2)
UNK	3 (1.2)	1 (1.0)	2 (1.4)
TOAST classification			
LAA	42 (17.1)	14 (14.0)	28 (19.3)
CAE	83 (33.9)	32 (32.0)	51 (35.2)
SAO	18 (7.3)	10 (10.0)	8 (5.5)
UND	102 (41.6)	44 (44.0)	58 (40.0)
Cholesterol (mmol/L)	4.92 ± 1.34	5.16 ± 1.38	4.76 ± 1.30
LDL (mmol/L)	3.09 ± 1.10	3.27 ± 1.05	2.94 ± 1.12
HDL (mmol/L)	1.28 ± 0.47	1.27 ± 0.43	1.29 ± 0.51
Creatinine (*μ*mol/L)	89.3 ± 54.4	86.9 ± 42.5	91.1 ± 61.8

Data presented as number (percentages), median (quintiles), or means ± standard deviation.

*Smoking history unknown in 61/245 (24.9%) of patients. LDL: low density lipoprotein cholesterol. HDL: high density lipoprotein cholesterol. LAA: large artery atherosclerosis, CAE: cardioembolic, SAO: small artery occlusion, UND: undetermined. TACI: total anterior circulation infarct, PACI: partial anterior circulation infarct, LACI: lacunar cerebral infarct, POCI: posterior circulation infarct, UNK: unknown site of the infarct.

**Table 2:** Distribution of calcifications by calcification type.

Vessel/type of calcif.	*N* (%)	Vessel area (mm^2^)	Focus Nr.	VL	VM	DL	DM	Extension (cont.)	Slice Nr. (median, quintiles)
VAs	236/245 (96.3)								
Right V.	155 (63.3)	71.2 ± 25.8							
Focal	141 (91.0)		1 (1-2)	59 (41.2)	56 (39.7)	42 (29.8)	70 (49.6)	73 (51.8)	1 (1-2)
Crescent	47 (30.3)		—	—	—	—	—	42 (89.4)	2 (1-2)
Circular	10 (6.4)		—	—	—	—	—	9 (90.0)	2 (1-2)
Left V.	186 (75.9)	79.8 ± 29.6							
Focal	168 (90.3)		1 (1-2)	79 (47.0)	79 (47.0)	46 (27.4)	84 (50.0)	92 (54.8)	2 (1-2)
Crescent	54 (29.0)		—	—	—	—	—	45 (83.3)	1 (1-2)
Circular	9 (4.8)		—	—	—	—	—	8 (88.9)	2 (1-2)

Basilar	42 (17.1)	77.6 ± 29.0		RV	RD	LV	LD		
Focal	42 (100.0)		1 (1-2)	24 (57.1)	14 (33.3)	15 (35.7)	11 (26.2)	17 (40.5)	1 (1-2)
Crescent	3 (7.1)		—	—	—	—	—	3 (7.1)	2 (1–3)
Circular	0 (0.0)		—	—	—	—	—	—	—

VAs: vertebral arteries; right V.: right vertebral; Left V.: left vertebral; focus Nr.: number of distinct focuses observed; VL: ventrolateral; VM: ventromedial; DL: dorsolateral; DM: dorsomedial; RV: right ventral; RD: right dorsal; LV: left ventral; LD: left dorsal; focal c.: focal calcifications; cont.: continuous extension of calcifications; data are *N* (per cent), median (quintiles 25%–75%), or mean ± standard deviation (st. dev.).

**Table 3:** Factors contributing to vertical dispersed intracranial calcifications in both vertebral and basilar arteries.

	Odds ratio (95% CI)	*P *
Age (years)	1.02 (0.99–1.05)	0.251
Male gender	2.71 (1.35–5.45)	0.005
Cholesterol mmol/L	0.98 (0.56–1.70)	0.933
LDL mmol/L	0.90 (0.46–1.75)	0.760
Diabetes history	2.27 (1.03–4.99)	0.040
Statin therapy	1.96 (0.92–4.17)	0.082
Smoking history*	0.98 (0.46–2.01)	0.951

LDL: low density lipoprotein, *positive smoking history versus never smoked + unknown history.
